# A Comparison of Job Satisfaction of Community Health Workers before and after Local Comprehensive Medical Care Reform: A Typical Field Investigation in Central China

**DOI:** 10.1371/journal.pone.0073438

**Published:** 2013-09-13

**Authors:** Hong Ding, Xin Sun, Wei-wei Chang, Liu Zhang, Xi-ping Xu

**Affiliations:** 1 School of Health Management, Anhui Medical University, Hefei, Anhui, China; 2 Department of Preventive Medicine, Wannan Medical College, Wuhu, Anhui, China; 3 Department of Hospital Infection Management Office, Wuhu Hospital of Traditional Chinese Medicine, Wuhu, Anhui, China; 4 Anhui Medical University, Biomedical Institute, Hefei, China; RAND Corporation, United States of America

## Abstract

**Background:**

The government of China promulgated new medical care reform policies in March 2009. After that, provincial-level governments launched new medical care reform which focusing on local comprehensive medical care reform (LCMR). Anhui Province is an example of an area affected by LCMR, in which the LCMR was started in October 2009 and implemented in June 2010. The objective of this study was to compare the job satisfaction (JS) of community health workers (CHWs) before and after the reform in Anhui Province.

**Methods:**

A baseline survey was carried out among 813 community health workers (CHWs) of 57 community health centers (CHCs) (response rate: 94.1%) and an effect evaluation survey among 536 CHWs of 30 CHCs (response rate: 92.3%) in 2009 and 2012 respectively. A self-completion questionnaire was used to assess the JS of the CHWs (by the job satisfaction scale, JSS).

**Results:**

The average scores of total JS and satisfaction with pay, contingent rewards, operating procedures and communication in the effect evaluation survey were statistically significantly higher than those of the baseline survey (*P*<0.05). The average score of satisfaction with promotion (2.55±1.008) in the effect evaluation survey was statistically significantly lower than that in the baseline survey (2.71±0.730) (*P*=0.002). In both surveys, the average scores of satisfaction with pay, benefits and promotion were statistically significantly lower than the others (all *P*<0.05).

**Conclusions:**

After two years’ implementation of the LCMR, CHWs’ total JS have a small improvement. However, CHWs have lower satisfaction in the dimensions of pay, promotion and benefits dimensions before and after the LCMR. Therefore, policy-makers should take corresponding measures to raise work reward of CHWs and pay more attention to CHWs’ professional development to further increase their JS.

## Introduction

Job satisfaction (JS) is defined as the positive personal perception towards work or work experiences [1]. Factors that influence JS comprise several aspects such as salary, working conditions, workload, workplace, career development, and the nature of the work [2]. In health service sectors, JS is highly associated with staffs’ turnover intention, quality and efficiency of services, and patients’ satisfaction [3–7]. Reports indicate doctors with higher job satisfaction are more likely to provide more satisfactory services and produce better therapeutic effect than those with lower ones [8].

Community health service organizations are primary health care organizations that provide public health services and primary medical services in urban China, which play important roles in the health-care network. China launched its Community Health Service (CHS) program in 1997 [9]. Since then, the community health service organizations have developed rapidly, and by the end of 2011, there were 7,861 community health centers (CHCs) with nearly 276,252 community health workers (CHWs) [10]. For a long time, health service managers mainly focused on how to improve the human resource supply and service capabilities of primary health care institutions, but failed to pay enough attention to the improvement the JS of CHWs. JS of CHWs is lower than those of employees working in urban hospitals in China [11]. Yin et al. conducted a survey of CHWs’ job satisfaction in three developed cities in China (Chengdu, Shenyang and Shanghai) and reported that JS of CHWs was low and 43.7% of CHWs had the turnover intention [12]. Another study conducted by Zhou et al. in five cities (Beijing, Suzhou, Jinan, Kunming and Xining) of China showed that only 30.6% of CHWs were satisfied with his or her working position and 58.0% of CHWs had the turnover intention [13]. In addition, several recent studies in China also reported similar results [14–19].

A report by Wang et al showed that 5.0-7.5% of CHWs left their professional positions in Anhui Province between 2007 and 2009, while their average age was 37.8 years and the average years of work seniority was 8.5 years [20]. The turnover of the young and middle-aged experienced staff in CHCs led to the instability of the organizational team, the reduction of the quality of service, the increase of cost for training manpower and the extension of cycle building organizational team.

The government of China promulgated new medical care reform policies in March 2009 [21]. After that, provincial-level governments launched new medical care reform focusing on local comprehensive medical care reform (LCMR), including "ensuring the basics(ensuring peoples^’^ access to basic health care services, basic public health services, essential medicines and basic medical insurance), strengthening the locals(strengthening construction of infrastructure, basic equipments and personnel ability of local health care institutions) and constructing the mechanisms(establishing local health care administration system and internal operation mechanism in local medical care institutions)".

Anhui Province is an example of an area affected by LCMR in China. Anhui Province, located in the central of China, has a total population of 690.2 million (urban population 230.5 million) with 16 cities in 2012. Anhui Province’s GDP per Capita in 2012 is about 28792 yuan (about 4561 dollars) and its economic development is moderate in China. In October 2009, Anhui was the first Chinese province to start LCMR, which included: (1) establishing competitive employment mechanisms and executing personnel management contracts; (2) establishing incentive mechanisms and conducting performance assessments; (3) establishing essential medicine systems and selling the essential medicines without increasing cost; (4) establishing financial guarantee mechanism to ensure that local health institutions will run normally [22].

In June 2010, the LCMR was implemented in 228 CHCs of Anhui Province. Currently, reports about LCMR efficacy on the JS of CHWs do not exist. The article is meant to assess the JS of CHWs before and after the LCMR in Anhui Province in order to provide evidence for improving the LCMR policy to increase the JS of CHWs.

## Methods

### Sample

Anhui Province is located in central China, with a total population of about 6600 CHWs in 228 urban CHCs in 2009. We conducted the baseline survey and the effect evaluation survey in 2009 and 2012 respectively. Before the survey, the checklist of CHCs was obtained from the child care and community health office of Anhui provincial health office. In the baseline survey which was conducted from August 2009 to October 2009, 57 CHCs were randomly chosen from 12 cities containing 188 CHCs in Anhui Province by using a systematic sampling method. In the effect evaluation survey which was conducted from June 2012 to July 2012, half of each city’s CHCs were randomly chosen for the baseline survey, and finally 30 CHCs were chosen randomly for the 2012 survey.

All subjects selected in our study worked in the fields of clinical, nursing, medico-technical, and public health services of the CHCs but leaders and administrative staffs were not included. Because of several sensitive problems in the questionnaires, the two surveys were completed anonymously. Each participant was informed by a verbal statement that participation in the study was voluntary and their privacy would be strictly protected. After receiving verbal consent from the participants for the conduct of the survey, a self-administered questionnaire survey was conducted among the CHWs. In 2009 survey, a total of 813 anonymous questionnaires were handed out to participants. Finally, 765 valid questionnaires were obtained, with an overall response rate of 94.1% (765/813). In 2012 survey, a total of 536 anonymous questionnaires were handed out to the participants. Finally, 495 valid questionnaires were available, with an overall response rate of 92.4% (495/536).

Ethical approval was granted by the Ethics Committee of Anhui Medical University.

### Questionnaire

The Job satisfaction scales (JSS), developed by Spector in 1985, was specifically developed for use in human service, public, or nonprofit organizations [23]. It includes 36 statements that elicit information on attitudes toward nine different job dimensions or aspects. The scales include satisfaction with pay (fairness, opportunities, frequency of raises), promotion (opportunities, fairness, frequency), supervision (level of competence, fairness, interest in subordinates), benefits (range of benefits, comparative value), contingent rewards (recognition, appreciation, rewards), operating procedures (rules and procedures, red tape, amount of work), coworkers (level of competence, friendliness), nature of work (interest, meaningfulness, enjoyment) and communication. When combined, these dimensions also constitute a measure of overall JS. Each dimension in the JSS is operationalized through four statements. Some statements are worded negatively and some positively. For example, the subscale for supervision is as follows: Item 3, my supervisor is quite competent in doing his/her job (positive); Item 12, my supervisor is unfair to me (negative); Item 21, my supervisor shows too little interest in the feelings of subordinates (negative); Item 30, I like my supervisor (positive).

The JSS uses a Likert-type rating scale with five agree-or-disagree response choices on each item of the 9 aspects. The scale ranged from 1 to 5 with 1 =very dissatisfied, 2 = dissatisfied, 3 = indifferent (neither satisfied nor dissatisfied), 4 = satisfied and 5 = very satisfied. These choices are approximately equally spaced psychologically in the response continuum according to the values generated by Spector (1976) and are scored 1 to 5, respectively, for positive statements. Negative statements were reversely scored. The subscale, the sum of responses for each four items ranges from 4 to 20 and the mean score of each subscale ranged from 1 (the highest possible degree of dissatisfaction) to 5 (the highest possible degree of satisfaction). The overall job satisfaction scale, the sum of responses for all 36 items ranges from 36 to 180 and the mean score ranged from 1 (the highest possible degree of dissatisfaction) to 5 (the highest possible degree of satisfaction). With acceptable reliability and validity, JSS has been widely used in many studies on various fields [24].

### Data analysis

Data were entered twice using EpiData 3.1 software and analyzed using SPSS13.0. Descriptive statistics, *t*-test, and Univariate Analysis of Variance were used in the statistical analysis. *P*≤0.05 were considered statistically significant.

## Results

### Demographic Characteristics

Table 1 showed the demographic characteristics of subjects in the baseline survey in 2009 and the effect evaluation survey in 2012. In the baseline survey, 813 CHWs took part in the investigation; accounting for 78.9% of the all health workers (the leaders and administrative staffs were excluded). After screening the invalid questionnaires, finally, 765 valid questionnaires were obtained (response rate is 94.1%), including 213 men (27.8%) and 552 women (72.2%). Among the subjects, the age group of less than 30 years and 30-39 years accounted for the highest proportion, 275 (36.0%) and 239 (31.2%), respectively. The highest proportion of job type were “clinic” and “nursing”, which was 317 (41.4%) and 262 (34.2%), respectively.

**Table 1:** Demographic characteristic of subjects.

**Variables**	**Group**	**2009 (N = 765,n[%])**	**2012 (N = 495,n[%])**	***χ*^*2*^**	***p***
Gender	Male	213 (27.8)	145 (29.3)	0.311	0.577
	Female	552 (72.2)	350 (70.7)		
Age	No more than 30	275 (36.0)	164 (33.1)	1.102	0.777
	30-39	239 (31.2)	164 (33.1)		
	40-49	157 (20.5)	105 (21.2)		
	over 49	94 (12.3)	62 (12.5)		
Job type	clinical	317 (41.4)	189 (38.2)	3.801	0.284
	Nursing	262 (34.2)	167 (33.7)		
	Medico-technique	84 (11.0)	54 (10.9)		
	public health	102 (13.3)	85 (17.2)		
Educational background	No medical educational background	8 (1.0)	0 (0.0)	6.127	0.106
	Secondary technical school graduates	281 (36.7)	195 (39.4)		
	Junior college graduates	354 (46.3)	228 (46.1)		
	Bachelor degree holders	122 (15.9)	72 (14.5)		
Professional titles	No technical title	102 (13.3)	62 (12.5)	1.863	0.601
	junior title	451 (59.0)	303 (61.2)		
	middle title	191 (25.0)	112 (22.6)		
	senior title	21 (2.7)	18 (3.6)		
Tenure (years)	1-5	214 (28.0)	142 (28.7)	1.874	0.866
	6-10	104 (13.6)	74 (14.9)		
	11-15	126 (16.5)	76 (15.4)		
	16-20	101 (13.2)	55 (11.1)		
	21-30	128 (16.7)	86 (17.4)		
	Over 30	92 (12.0)	62 (12.5)		

In the effect evaluation survey, 536 CHWs took part in the investigation; accounting for 86.7% of the all health workers (the leaders and administrative staffs were excluded). After screening the invalid questionnaires, finally, 495 valid questionnaires were available (response rate is 92.4%), There were no significant differences in gender, age, job type, educational background, professional titles and tenure between the two surveys (*p*>0.05) (Table 1).

### Job satisfaction comparison

After two years’ implementation of the LCMR, the average score of total JS of the CHWs was 3.13, which was statistically significantly higher than the baseline survey (average score: 3.06) (*P*=0.029). The average score of satisfaction with pay, contingent rewards, operating procedures and communication in the effect evaluation survey were statistically significantly higher than the baseline survey (*P*<0.05, Table 2). However, the average score of satisfaction with promotion (2.55±1.008) was statistically significantly lower than the baseline survey (2.71±0.730) (*P*=0.002). There were no significant differences in the dimensions of supervision, benefits, coworkers and nature of work between the two surveys (*p*>0.05) (Table 2).

**Table 2:** CHWs’ job satisfaction scores of the two surveys.

	**2009 $\bar{\mathrm{(\chi}}$±s)**	**2012 $\bar{\mathrm{(\chi}}$±s)**	***t***	***p***
Pay	2.50±0.715	2.72±1.037	-4.134	0.000
Promotion	2.71±0.730	2.55±1.008	3.062	0.002
Supervision	3.44±0.753	3.45±0.994	-0.266	0.790
Benefits	2.56±0.768	2.48±1.121	1.418	0.157
Contingent Rewards	2.98±0.732	3.37±0.977	-7.637	0.000
Operating Procedures	2.91±0.581	3.07±0.928	-3.387	0.001
Coworkers	3.68±0.518	3.63±0.847	1.053	0.293
Nature of Work	3.63±0.670	3.55±1.013	1.460	0.145
Communication	3.12±0.730	3.37±1.105	-4.432	0.000
Overall	3.06±0.497	3.13±0.643	-2.191	0.029

In the baseline survey, the three lowest-score dimensions ranked successively as follows: pay (2.50±0.715), benefits (2.56±0.768) and promotion (2.71±0.730). After the LCMR, the rank was benefits (2.48±1.121), promotion (2.55±1.008) and pay (2.72±1.037). CHWs had the lowest satisfaction in the dimensions of pay, promotion and benefits dimensions whether before or after the LCMR.

### Total JS comparison adjusted by age, educational background, professional titles and tenure

As we all know, JS may be influenced by age, educational background, professional titles and tenure. By using Univariate Analysis of Variance adjusted by age, educational background, professional titles and tenure, the average score of total job satisfaction in 2012 survey was (3.13±0.025), which was statistically significantly higher than the baseline survey (average score: 3.06±0.020) (*P*=0.021) (Table 3).

**Table 3:** The outcome of the univariate analysis of variance between the two surveys.

Year	Total score	*F/p*
2009 $\bar{\mathrm{(\chi}}$±s)	3.06±0.020	5.347/0.021
2012 $\bar{\mathrm{(\chi}}$±s)	3.13±0.025	

Adjusted age, Educational background, Professional titles, Tenure (years), R Squared =0.014 (Adjusted R Squared =0.010)

## Discussion

Staff job satisfaction in CHCs has important implications for sustainable development of basic healthcare in China, but health decision-makers failed to pay enough attention to job satisfaction of grass roots medical workers for a long time [25]. Past experience showed that the CHCs cannot survive and thrive without a team of dedicated workers equipped with adequate medical skills [14]. As primary health service providers, policy-makers need take measures to improve CHCs’ environment and conditions to operate efficiently and to meet rational demands of their employees.

In order to assess the JS of CHWs before and after the LCMR in Anhui Province, JSS was used to measure CHWs’ job satisfaction scores, the score of total job satisfaction and each dimensions ranged from 1 to 5 with 1 =very dissatisfied, 2 = dissatisfied, 3 = indifferent (neither satisfied nor dissatisfied), 4 = satisfied and 5 = very satisfied. The LCMR was implemented in Anhui Province in June 2010. Two years later, we found that the average score of total JS of the CHWs in 2012 survey was 3.13, which was statistically significantly higher than the average score (3.06) in 2009 baseline survey. After evaluating the effect of reform from the perspective of personnel JS, a series of measures of reform have realized certain achievements. However, there was only a small improvement in the total job satisfaction of CHWs.

After the reform, the scores of JS with contingent rewards was higher than the baseline survey in 2009. The mean score of JS had been increased from 2.98 to 3.37, moreover, the scores of JS with pay and operating procedures also had a small raise, which may be attributed related to some policy measures (for example: guarantee of the CHWs wages by government finance, implementing performance management, the regulations of the management and technical process, etc.). It is a puzzling problem that the score of JS with promotion was lower than the baseline survey, although policy-makers designed a series of policies and measures(such as the access system of the CHWs, the competitive mechanism for jobs and the standard system for personnel training, etc) to secure stable occupation developments for CHWs. Based on the information in field interviews, the main causes might be: ① personnel training system is mainly focused on knowledge training, while skills training did not meet the needs of the CHWs; ② the high requirement of promotion conditions (such as requirements for the CHWs to publish a number of academic papers and undertake a certain level scientific research project) made it difficult for the CHWs to get higher professional titles.

In this study, we also found that the CHWs had higher scores of JS in the dimensions of coworkers, nature of work and supervision before and after the LCMR, thus, it suggests that most workers get along well with their colleagues, consider their jobs to be of importance, and identify with the leadership style, which provide a good foundation for the development of CHS. However, CHWs had lower scores of JS in the dimensions of pay, promotion, and benefits compared with others both in the baseline survey and the effect evaluation survey. Many previous studies also showed that CHWs felt most dissatisfied with welfare, pay, and promotion opportunity [26–29]. Therefore, welfare, pay, and promotion opportunity are still critical factors that influence JS of CHWs.

This is the first study to evaluate the effectiveness of the LCMR from the perspective of JS of CHWs. So there may be some limitations in the study. First, the study evaluates the effectiveness of the LCMR only from the perspective of JS of CHWs while not from the perspective the actual benefits of residents and JS of patients. Second, LCMR was implemented in Anhui province in June 2010, two years later, and effect evaluation survey was conducted in 2012. It may be that the effect of reform has not yet been reflected in a short duration, which may be resulted in small changes of scores between the two studies. Third, all information was obtained from a self-reported questionnaire and the response bias was thus unavoidable.

In conclusion, after two years’ implementation of the LCMR, CHWs’ total job satisfaction have a small improvement. CHWs have the lowest satisfaction in the dimensions of pay, promotion and benefits dimensions before and after the LCMR. Policy-makers need systematically evaluate the CHWs’ demand for their professional development and expectations. At the same time, the corresponding policies and their implementation process should be systematically evaluated, so that the policies could be revised and improved timely, which can increase the job satisfaction of the CHWs and contribute to the overall quality of CHS.
